# Personalized Clinical Workflow for Compression Therapy in Post-Burn Hypertrophic Scars Using 3D Imaging and Computational Modeling: A Feasibility Study

**DOI:** 10.3390/healthcare14152438

**Published:** 2026-08-06

**Authors:** Tomáš Demčák, Erik Eliáš, Július Uchnár, Katarína Dudová, Bibiána Ondrejová, Monika Michalíková, Lucia Bednarčíková, Branko Štefanovič, Jozef Živčák, Peter Lengyel

**Affiliations:** 1 Department of Burns and Reconstructive Surgery, AGEL Hospital Košice-Šaca, 04015 Košice, Slovakia; tomas.demcak@nke.agel.sk (T.D.); erik.elias@nke.agel.sk (E.E.); julius.uchnar@nke.agel.sk (J.U.); peter.lengyel@nke.agel.sk (P.L.); 2 Department of Biomedical Engineering and Measurement, Faculty of Mechanical Engineering, Technical University of Košice, 04200 Košice, Slovakia; bibiana.ondrejova@tuke.sk (B.O.); monika.michalikova@tuke.sk (M.M.); lucia.bednarcikova@tuke.sk (L.B.); branko.stefanovic@tuke.sk (B.Š.); jozef.zivcak@tuke.sk (J.Ž.)

**Keywords:** burn scars, compression therapy, three-dimensional imaging, personalized medicine

## Abstract

**Highlights:**

**What are the main findings?**

- A structured two-phase clinical workflow integrating scar assessment, 3D surface imaging, and stratification for personalized compression therapy was successfully implemented.
- Three-dimensional imaging was feasible in all evaluated scars and provided enhanced visualization of scar morphology and surface topography compared with standard photography.

**What are the implications of the main findings?**

- Objective scar stratification may support more consistent identification of patients suitable for personalized compression therapy.
- Patient-specific compression matrices may improve anatomical fit and spatial control of pressure delivery, although their clinical effectiveness requires longitudinal validation.

**Abstract:**

**Background:** Hypertrophic scarring remains a common and clinically challenging consequence of burn injury. Although compression therapy is widely used, its effectiveness is limited by variability in pressure delivery, lack of personalization and inconsistent treatment protocols. **Methods:** This prospective single-centre feasibility study included adult patients with post-burn scars following deep partial-thickness or full-thickness injuries. The study was designed as a two-phase workflow. In Phase I, scars were documented using standardized photography and 3D surface scanning and were evaluated using the Vancouver Scar Scale (VSS). Scars were stratified based on a predefined threshold (VSS >6) for progression to intervention. In Phase II, patient-specific compression matrices will be designed from 3D surface data and fabricated using biocompatible material. Computational modeling using finite element analysis (FEA) will be applied to simulate pressure distribution. Follow-up is planned at 1, 3, and 6 months, with the VSS as the primary outcome measure. **Results:** A total of 27 scars were included. The median VSS score was 7 (IQR 5–8). Based on stratification, 14 scars (51.9%) were eligible for personalized compression therapy. Three-dimensional imaging was feasible in all cases and allowed for improved qualitative visualization of scar morphology compared to standard photography. Preliminary findings support the applicability of the proposed workflow, while longitudinal outcome analysis is ongoing. **Conclusions:** This feasibility study presents and evaluates a clinical workflow combining 3D imaging, objective scar stratification, and personalized compression therapy. This approach provides a structured framework for personalized compression therapy, while its clinical effectiveness remains to be established. Further studies are required to evaluate long-term outcomes.

## 1. Introduction

Burn injuries remain a significant cause of long-term morbidity, with hypertrophic scarring representing one of their most challenging sequelae. Hypertrophic scars develop in more than 70% of burn survivors following deep dermal and full-thickness burns, substantially affecting function, appearance, and quality of life [1,2]. These scars typically develop within weeks after re-epithelialization and are characterized by increased thickness, erythema, reduced pliability, pruritus, and a tendency toward contracture, often leading to functional and aesthetic impairment [3,4]. Unlike keloids, hypertrophic scars remain confined to the boundaries of the original wound and may partially regress over time, whereas keloids extend beyond the original wound margins [5]. Their pathophysiology involves excessive fibroblast activity and disorganized collagen deposition, resulting in altered biomechanical properties of the skin and reduced quality of life.

Conservative scar management remains the cornerstone of post-burn care and relies on a multimodal approach, including pressure therapy, silicone-based products, massage, and hydration [6]. Pressure garments exert continuous mechanical compression, which is thought to reduce local perfusion and fibroblast activity, thereby limiting collagen synthesis and scar hypertrophy [7]. Mechanical forces regulate fibroblast activity through mechanotransduction pathways, promoting persistent myofibroblast activation, dysregulated TGF-β signaling, and excessive extracellular matrix deposition, all of which contribute to hypertrophic scar formation [1,3].

Despite its widespread use, the clinical efficacy of pressure therapy remains controversial. While some studies report improvements in scar thickness, erythema, and pliability, others show inconsistent results, likely due to variability in treatment protocols, pressure levels, and patient adherence [7,8]. Optimal parameters, including pressure magnitude and treatment duration, are not clearly defined, although pressures of 20–25 mmHg and prolonged wear are commonly recommended [6,9,10]. Furthermore, the actual interface pressure delivered by conventional compression garments is rarely measured in routine clinical practice and may vary considerably according to anatomical location and garment fit. This variability highlights the need for more objective and reproducible methods of scar assessment and treatment planning, enabling better stratification of patients and more targeted therapeutic interventions.

One of the key contributors to this variability is the inability of conventional compression therapy to deliver uniform and patient-specific pressure. Anatomical variability and scar location often result in inconsistent pressure distribution, particularly in complex regions such as the face, neck, and joints [7]. In addition, standard garments are typically based on approximate measurements rather than precise three-dimensional geometry.

Recent advances in three-dimensional (3D) scanning and additive manufacturing offer a promising solution to these limitations. Compared with conventional photography and manual measurements, high-resolution 3D surface imaging enables objective scar assessment, precise anatomical modeling, and patient-specific treatment planning. Three-dimensional surface scanning further facilitates the design of individualized compression devices tailored to patient anatomy, potentially improving pressure distribution, treatment precision, comfort, and adherence [11,12]. Therefore, this study aimed to develop and evaluate a personalized approach to compression therapy for post-burn hypertrophic scars using three-dimensional scanning and customized compression matrix fabrication. To our knowledge, this is the first study to integrate standardized scar stratification, three-dimensional surface imaging, and patient-specific compression design into a single clinical workflow.

## 2. Methods

This prospective single-centre clinical study was conducted at the Department of Burns and Reconstructive Surgery, AGEL Hospital Kosice-Saca, in collaboration with the Department of Biomedical Engineering and Measurement, Faculty of Mechanical Engineering, Technical University of Kosice (TUKE). The study was designed as a two-phase workflow integrating three-dimensional (3D) imaging into scar assessment and subsequent individualized compression therapy. Adult patients (>18 years) with post-burn scars following deep partial-thickness (grade IIb) or full-thickness (grade III) burns requiring surgical treatment were eligible for inclusion. Only scars resulting from burn injury were included. Patients were enrolled after complete wound healing and completion of acute burn treatment, including dermo-epidermal grafting. Scar assessment was performed at the first outpatient visit after complete epithelialization, which typically occurred approximately 4–8 weeks after burn injury. No minimum or maximum scar size was predefined for study inclusion. All eligible post-burn scars suitable for standardized three-dimensional imaging were considered. Patients with epilepsy were excluded as a precaution because structured-light projection used during optical scanning may theoretically trigger photosensitive seizures in susceptible individuals. For the interventional phase, only scars located on anatomically suitable regions (flat or mildly curved surfaces) were considered, whereas scars over highly mobile joints, deep anatomical concavities, or areas where stable positioning of the compression matrix could not be ensured were excluded. The study was approved by the Institutional Ethics Committee of AGEL Hospital Kosice-Saca (protocol No. 17-2023). Written informed consent was obtained from all participants.

### 2.1. Phase I: Imaging and Workflow Development

In the first phase, a standardized protocol for scar documentation and three-dimensional (3D) imaging was established and validated. Each scar was documented using standardized clinical photography and 3D surface scanning. Two handheld scanners were used: the Artec Eva (Artec 3D, Luxembourg) as a structured-light reference system and the Revopoint Miraco (Revopoint 3D Technologies Inc., Shenzhen, China) as a low-cost portable alternative.

Scanning was performed under standardized clinical conditions by one of two trained operators following an identical acquisition protocol. Patients were positioned in a standardized posture according to the anatomical location of the scar to ensure reproducibility during repeated examinations. The Artec Eva scanner was operated at a working distance of approximately 0.5–0.7 m from the skin using the manufacturer’s recommended acquisition settings (3D resolution up to 0.2 mm, 3D accuracy up to 0.1 mm, and a capture rate of up to 16 frames/s). Ambient lighting conditions were maintained at approximately 500 lx while avoiding direct reflections. The average acquisition time was approximately 15 min, depending on the size and anatomical complexity of the scanned region.

Three-dimensional reconstruction was performed in Artec Studio 13 (Artec 3D, Luxembourg) using the standard processing workflow, including automatic registration, global registration, noise removal, and mesh fusion. No additional smoothing filters were applied. Each reconstructed model was visually inspected to verify complete surface coverage, successful registration, and the absence of tracking artefacts or missing regions. Scans that did not meet these quality criteria were repeated. Quantitative mesh quality metrics, such as mesh error or point density, were not evaluated as the primary objective of this study was workflow feasibility rather than technical validation of the scanning systems. This phase aimed to establish a reproducible imaging workflow, compare the feasibility and output quality of different scanning systems, and enable objective capture of scar morphology.

### 2.2. Scar Assessment

Following imaging, all scars were evaluated using the Vancouver Scar Scale (VSS) [13], which includes vascularity, pigmentation, pliability, and height. The VSS was selected as the primary assessment tool due to its simplicity, widespread use in burn scar assessment, and suitability for routine clinical practice. The Patient and Observer Scar Assessment Scale (POSAS) [14] was not applied in this study to maintain a streamlined and reproducible evaluation process. Each scar was assessed independently by two physicians experienced in burn scar evaluation and familiar with routine VSS application. Before study assessment, both evaluators reviewed the VSS domains and scoring criteria to ensure consistent interpretation of the scale. Discrepancies between evaluators were resolved by consensus discussion. A third evaluator was not required, as all disagreements were resolved by consensus. Scars were stratified based on total VSS score, with a predefined threshold of >6 indicating eligibility for the interventional phase. The threshold (VSS > 6) was selected to identify clinically relevant moderate-to-severe hypertrophic scars requiring intervention. This cutoff was based on the distribution of VSS scores observed in the preliminary cohort, where the median total VSS score was 7, and was considered clinically appropriate for distinguishing scars with relevant hypertrophic features from milder scars. Interobserver agreement was not formally quantified because of the feasibility-study design and the exploratory nature of the workflow.

To minimize selection bias, eligible patients were enrolled consecutively according to the predefined inclusion criteria. VSS evaluators were not formally blinded to eligibility for the interventional phase as patient stratification was determined directly by the VSS score together with anatomical suitability, making blinding impractical within the study design. Consistency between the two three-dimensional scanning systems was ensured by using a standardized image acquisition protocol for both devices. In addition, scanner concordance has previously been evaluated in our published methodological study, which demonstrated comparable three-dimensional visualization of burn wounds using the Artec Eva and Revopoint Miraco systems [15].

### 2.3. Phase II: Personalized Compression Therapy

**Figure 1:**
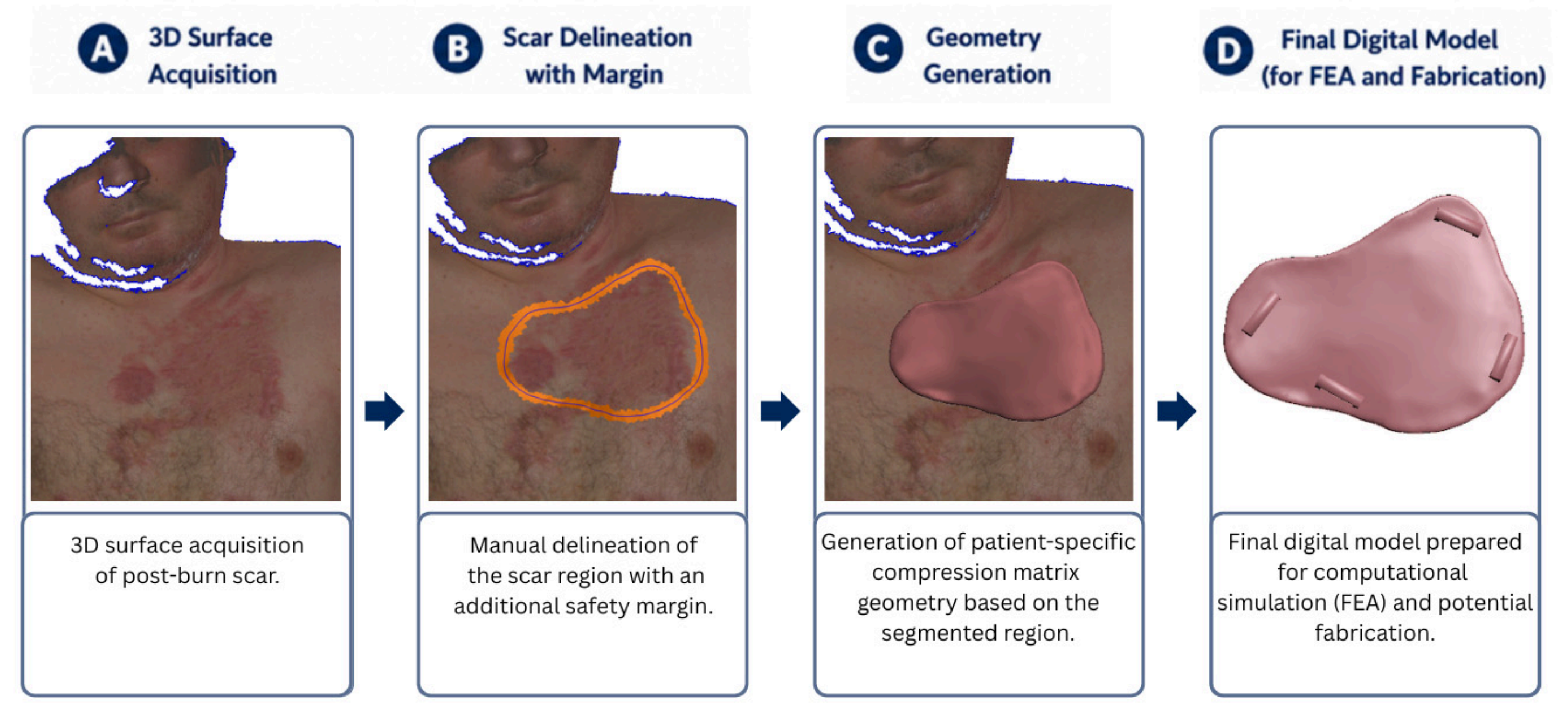
Digital workflow for patient-specific compression matrix design.

For scars meeting inclusion criteria, 3D surface data will be used to design individualized compression matrices in collaboration with the TUKE. Patient-specific compression matrices were digitally designed using Autodesk Fusion 360 (Autodesk Inc., San Francisco, CA, USA) based on three-dimensional surface data derived from optical scanning. The scar region was delineated with an additional margin extending into surrounding healthy tissue, and a conforming orthosis geometry was generated to ensure adequate contact and anatomical adaptation. The design process allowed for controlled shaping of the compression surface and incorporation of fixation features to improve stability during wear. The digital modelling workflow is illustrated in Figure 1.

Medical-grade polylactic acid (PLA) filament certified for skin-contact applications will be used for fabrication of the personalized compression matrices. PLA was selected because of its biocompatibility, dimensional stability, ease of additive manufacturing, and suitability for rapid patient-specific prototyping. The design is intended to provide localized and sustained compression. Application will be limited to anatomically suitable regions to ensure stable positioning and reproducible pressure delivery. Patients will be instructed to wear the personalized compression matrix for up to 23 h per day, removing it only for personal hygiene and skin inspection. Compression therapy is planned for a period of six months, with reassessment at 1-, 3-, and 6-month follow-up visits. Matrix adjustment or redesign may be performed if clinically indicated. The target pressure range was defined as 20–25 mmHg based on current clinical recommendations. The interventional phase is ongoing and not reported in the present manuscript.

### 2.4. Computational Modeling (FEA)

Patient-specific three-dimensional (3D) surface models derived from optical scanning may be used for computational simulation of pressure distribution using finite element analysis (FEA). Three-dimensional scar and compression matrix geometries will be imported into FEBio Studio 4.13 (University of Utah, Salt Lake City, UT, USA), an open-source software platform for biomechanical finite element modeling. The compression matrix will be modeled as a PLA-based elastic structure, while the underlying skin will be represented as a simplified deformable soft-tissue model using material properties reported in the literature. Contact interactions between the compression matrix and the skin surface will be defined, and displacement-controlled loading will be applied to simulate clinical fixation of the device. Simulations will be performed to estimate relative pressure distribution across the scar surface, with the design objective of achieving the clinically recommended interface pressure of approximately 20–25 mmHg while minimizing localized pressure peaks. The primary purpose of FEA will be to support iterative optimization of the compression matrix geometry before fabrication. The simulations are intended as an exploratory design-support tool and do not represent validated clinical pressure measurements.

During the interventional phase, we plan to use a suitable thin flexible pressure measurement system when available for interface pressure validation. Direct pressure measurements will be used to assess the pressure distribution between the personalized compression matrix and the scar surface and to compare the achieved interface pressure with the target therapeutic range of 20–25 mmHg. If required, the geometry or fixation of the compression matrix may be modified to improve pressure distribution. Until direct pressure measurements become available, finite element analysis will be used as an exploratory tool to estimate relative pressure distribution and support iterative optimization of individualized compression matrix design.

### 2.5. Follow-Up and Planned Outcome Assessment

**Figure 2:**
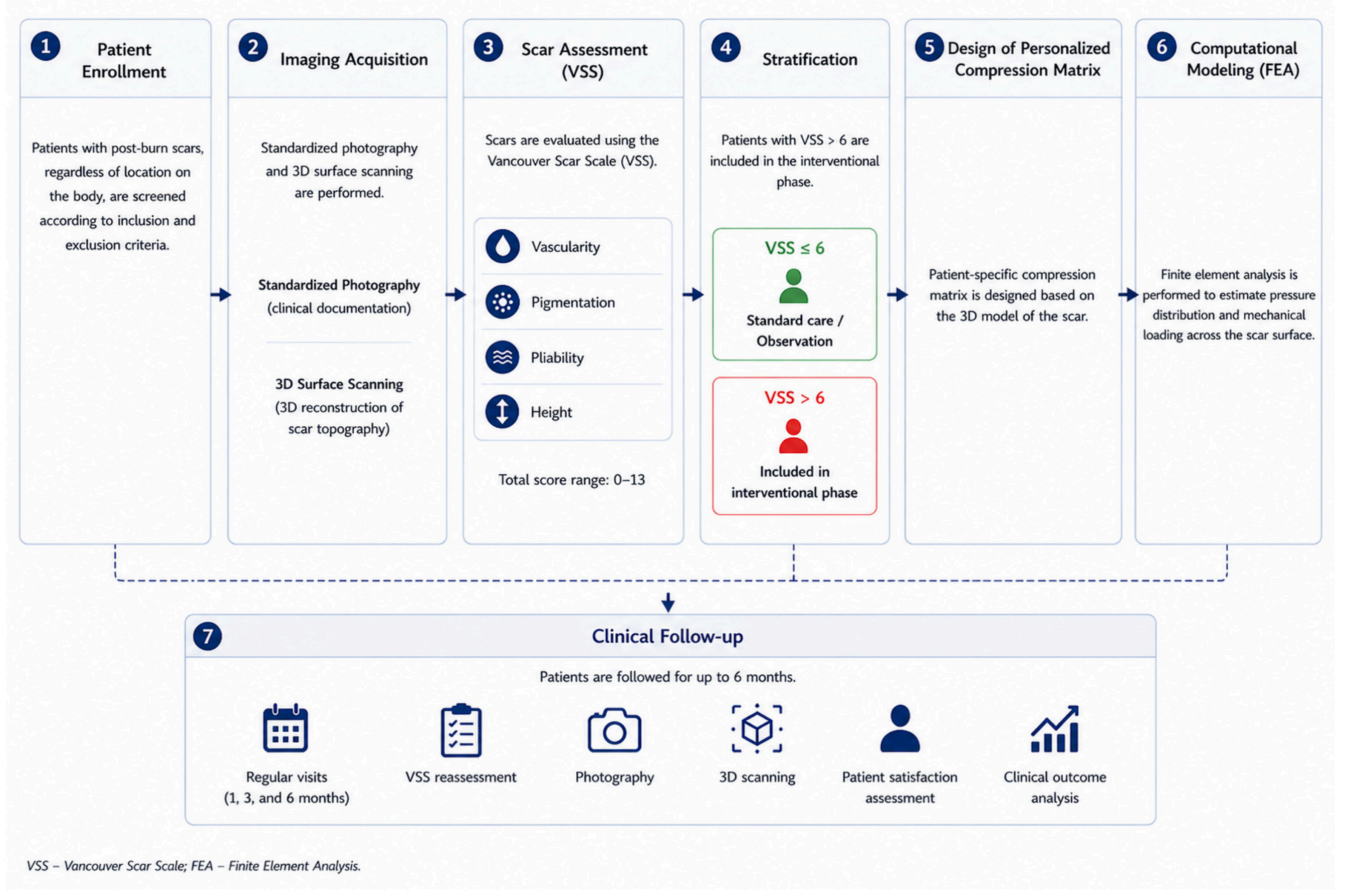
Overview of the clinical workflow integrating three-dimensional (3D) imaging, scar assessment, and personalized compression therapy. Patients with post-burn scars undergo standardized photography and 3D surface scanning, followed by evaluation using the Vancouver Scar Scale (VSS) and stratification for personalized compression therapy.

Patients indicated for compression therapy are scheduled for follow-up at 1 month, 3 months, and 6 months after treatment initiation. At each visit, scars will be evaluated using the Vancouver Scar Scale (VSS), standardized clinical photography, and three-dimensional surface scanning. Standardized photographs will be obtained using identical patient positioning, lighting conditions, camera settings, and imaging distance whenever feasible. The primary outcome will be the change in total VSS score from baseline to the 6-month follow-up. Secondary outcomes will include changes in individual VSS domains (vascularity, pigmentation, pliability, and height), qualitative and semi-quantitative assessment of scar morphology using three-dimensional surface models, feasibility of the scanning workflow (including acquisition time), and treatment adherence. Treatment adherence will be assessed at each follow-up visit based on patient self-report and clinical interview. These analyses are planned and are not yet available at the time of manuscript preparation (Figure 2).

### 2.6. Statistical Analysis

The study is designed as a feasibility and proof-of-concept clinical investigation. Baseline characteristics are reported using descriptive statistics. Continuous variables are expressed as medians and interquartile ranges (IQRs), and categorical variables as counts and percentages.

Longitudinal analysis of VSS changes will be performed after completion of follow-up. A *p*-value < 0.05 will be considered statistically significant. Comparisons between time points will be performed using appropriate non-parametric tests. At the time of manuscript preparation, the interventional phase is ongoing and longitudinal outcome data are not yet available.

## 3. Results

The primary outcome of Phase I was the feasibility of three-dimensional (3D) imaging and implementation of the proposed clinical workflow. 3D surface imaging was successfully performed in all included cases with no acquisition failures observed. The average time required for image acquisition was approximately 15 min per scan under routine clinical conditions.

### 3.1. Study Population

**Figure 3:**
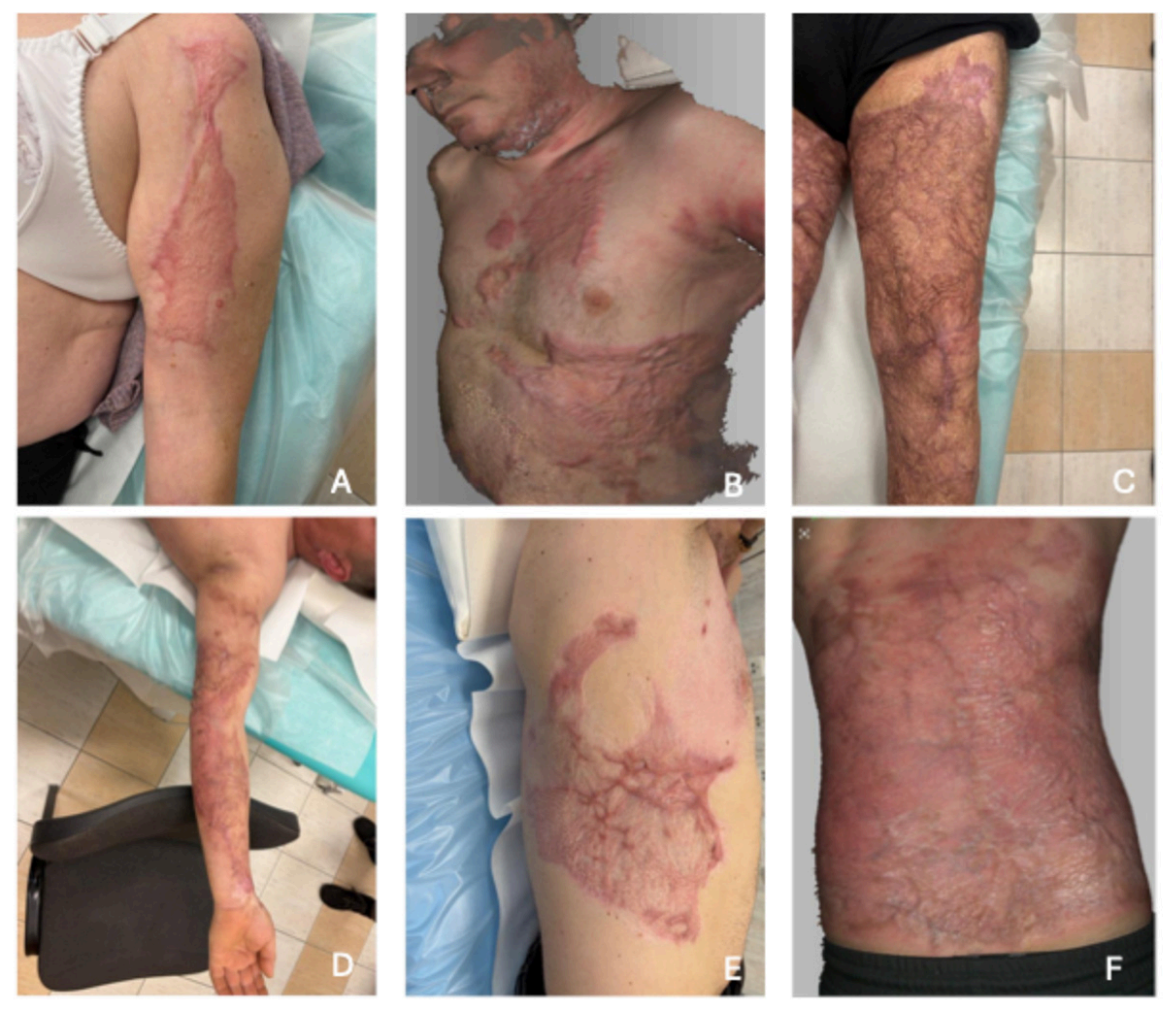
Representative post-burn scars of patients included in the study cohort. (**A**) Upper-arm scar with linear hypertrophic component and surrounding erythema. (**B**) Extensive anterior trunk scar with irregular distribution and mixed hypertrophic features. (**C**) Lower-extremity scar demonstrating pronounced hypertrophy with mesh graft pattern. (**D**) Forearm scar with longitudinal distribution and areas of variable pliability. (**E**) Lateral-trunk scar with heterogeneous pigmentation and irregular borders following grafting. (**F**) Posterior-trunk scar with diffuse hypertrophic changes and altered surface texture.

A total of 27 scars from adult patients were included in the analysis. The cohort consisted of 18 male (66.7%) and 9 female (33.3%) patients. All evaluated scars were post-burn following deep partial-thickness or full-thickness injuries. Most patients were assessed approximately 6–8 weeks after injury, corresponding to the early post-grafting phase of scar maturation. Table 1 summarizes the baseline characteristics of the study cohort. Representative post-burn scars of patients included in the study cohort are shown in Figure 3.

### 3.2. Phase I: Feasibility of 3D Scanning and Imaging Comparison

Three-dimensional surface imaging was successfully performed in all included cases using both Artec Eva and Revopoint Miraco. The Artec Eva system provided superior surface detail and more accurate delineation of scar margins, while the Revopoint Miraco device demonstrated lower optical fidelity but remained sufficient for capturing overall scar morphology. In anatomically simple regions, both systems produced comparable geometric outputs [12]. Compared to standard photography, 3D scanning enabled objective visualization of scar elevation and surface irregularities, which were not reliably captured using two-dimensional imaging. The median total VSS score across the cohort was 7 (IQR 5–8). Based on predefined stratification, 13 scars (48.1%) were classified as VSS ≤ 6, while 14 scars (51.9%) were classified as VSS > 6. Scars with higher VSS scores were primarily characterized by increased pliability and height, consistent with hypertrophic scar features. Table 2 presents the distribution of Vancouver Scar Scale (VSS) subdomain scores in the study cohort.

### 3.3. Phase II: Selection for Compression Therapy

A total of 14 scars (51.9%) met the predefined threshold (VSS > 6) and were considered eligible for progression to the interventional phase. Further selection for compression matrix application is ongoing and depends on anatomical suitability (e.g., exclusion of scars over joints or highly irregular regions). At this stage, these patients represent the target cohort for individualized compression therapy. Follow-up assessments and outcome measures are described in Section 2; analysis of these outcomes is ongoing and final clinical outcome data will be reported upon study completion.

### 3.4. 3D Morphological Assessment (Planned)

Three-dimensional imaging will be used for longitudinal comparison of scar topography. Changes in scar elevation, surface regularity, and overall morphology will be assessed across follow-up visits, enabling objective documentation of treatment response. Safety evaluation will include monitoring of adverse events such as skin irritation, discomfort, or pressure-related complications. Patient adherence will be assessed based on reported daily usage. The feasibility of integrating patient-specific compression matrices into routine clinical practice will be evaluated in terms of workflow, reproducibility, and patient compliance.

These cases illustrate the variability in scar morphology, anatomical location, and surface characteristics relevant for three-dimensional (3D) assessment and individualized compression design. Clinical outcome data from the interventional phase are not yet available and will be reported in future analyses.

## 4. Discussion

This feasibility study proposes a structured, workflow-based approach to post-burn scar management integrating 3D imaging, objective scar assessment, and personalized compression therapy. The proposed two-phase design allows for systematic transition from standardized scar documentation and stratification to targeted, patient-specific intervention.

Pressure therapy remains a cornerstone of conservative scar management; however, its clinical effectiveness has been inconsistently reported. Variability in treatment protocols, pressure magnitude and patient adherence contributes to heterogeneous outcomes, limiting reproducibility across studies [6,7,8,9,10]. In addition, conventional compression garments are typically based on approximate measurements and are unable to deliver uniform pressure across irregular scar surfaces. As a result, localized under- or over-compression may occur, potentially reducing therapeutic efficacy and contributing to patient discomfort.

The present study addresses these limitations by introducing a structured workflow combining objective scar assessment with individualized compression design. The use of the Vancouver Scar Scale (VSS) enabled pragmatic and reproducible stratification of scars, allowing for identification of patients most likely to benefit from intervention. By selecting a predefined threshold (VSS > 6), the study focuses on clinically relevant hypertrophic scars, aligning therapeutic effort with disease severity. While more comprehensive tools such as POSAS exist, the use of VSS in this context supports feasibility and scalability in routine clinical practice.

A key innovation of this approach lies in the integration of 3D surface imaging into treatment planning. Unlike conventional two-dimensional assessment, 3D scanning enables accurate capture of scar topography, facilitating the design of compression matrices tailored to individual anatomy. This allows for more precise adaptation to local curvature and surface irregularities, which are critical determinants of pressure distribution. In contrast to standard garments, patient-specific matrices may enable more controlled and anatomically adapted mechanical loading across the scar surface.

The workflow begins with 3D surface acquisition of the post-burn scar (A), followed by manual delineation of the scar with a safety margin (B). A patient-specific compression matrix geometry is then generated based on the segmented region (C). The final digital model is subsequently prepared for computational simulation using finite element analysis (FEA) and potential fabrication (D).

Recent evidence suggests that the effectiveness of pressure therapy is influenced not only by pressure magnitude but also by its spatial distribution and consistency over time [6,7,10]. In this context, patient-specific, geometry-driven compression design represents rational advancement as it enables adaptation to individual scar morphology and may improve the consistency of mechanical loading across the treated area.

Furthermore, computational modeling represents an important step toward objective optimization of compression therapy. Finite element analysis (FEA) offers the possibility to simulate pressure distribution and identify areas of uneven loading prior to clinical application. Although not directly validated by in vivo measurements, this approach provides insight into relative pressure distribution patterns and may guide optimization of compression device design. Similar computational approaches have been successfully used to model pressure distribution and prevent tissue damage in diabetic foot ulcers, providing a theoretical framework for optimization of pressure distribution in scar management [16,17,18,19].

From a clinical perspective, this combined strategy may improve treatment precision, enhance reproducibility and potentially increase patient adherence through better device fit and comfort. However, patient adherence to conventional compression therapy remains a significant challenge due to discomfort, skin irritation, and the need for prolonged daily use.

The use of anatomically adapted compression matrices may be particularly advantageous in regions where conventional garments are less effective, although this study intentionally focused on simpler anatomical areas to ensure initial feasibility.

Several limitations should be acknowledged. First, the study represents an early-phase feasibility investigation, and longitudinal outcome data are currently pending. Second, pressure distribution in biological tissues is influenced by complex and patient-specific biomechanical properties, which may not be fully captured by computational models. The absence of direct pressure measurements limits validation of the computational model, and therefore FEA results should be interpreted as estimations of relative pressure distribution rather than absolute values. Third, the exclusion of scars located over joints or highly complex anatomical regions limits generalizability but was necessary to establish a controlled and reproducible workflow. Finally, the relatively small sample size and the single-center design limits the generalizability of the findings.

Despite these limitations, the proposed approach provides a foundation for a more individualized and objective paradigm in scar management. Future studies should focus on validating the relationship between measured and simulated pressure and clinical outcomes, as well as expanding the application to more complex anatomical regions. This study primarily reports feasibility and workflow development, while the clinical effectiveness of the proposed intervention remains to be established. At present, the proposed workflow should be considered as a feasibility framework rather than an evidence-based therapeutic approach.

## 5. Conclusions

This feasibility study presents a standardized workflow integrating three-dimensional surface imaging, clinical scar assessment, and personalized compression matrix design for the management of post-burn hypertrophic scars. The proposed approach demonstrates the technical feasibility of combining objective scar documentation with patient-specific treatment planning. The ongoing interventional phase will determine whether this workflow can improve pressure delivery, scar outcomes, and clinical applicability in routine burn care.

## Figures and Tables

**Table 1:** Baseline Characteristics of the Study Cohort.

Variable	Value
Number of patients/scars	27
Male sex, *n* (%)	18 (66.7%)
Female sex, *n* (%)	9 (33.3%)
Age, median (IQR), years	42 (29–58)
Time from injury to assessment, median (IQR), weeks	8 (6–12)
Burn depth	
– Deep partial-thickness (IIb), *n* (%)	16 (59.3%)
– Full-thickness (III), *n* (%)	11 (40.7%)
Grafted scars, *n* (%)	27 (100%)
Scar location	
– Upper extremity, *n* (%)	11 (40.7%)
– Lower extremity, *n* (%)	9 (33.3%)
– Trunk, *n* (%)	7 (25.9%)
VSS total score, median (IQR)	7 (5–8)
VSS ≤ 6, *n* (%)	13 (48.1%)
VSS > 6, *n* (%)	14 (51.9%)

**Table 2:** Vancouver Scar Scale Subdomain Scores.

Parameter	Median (IQR)
Vascularity	2 (1–3)
Pigmentation	2 (1–2)
Pliability	3 (2–4)
Height	2 (1–2)
Total VSS	7 (5–8)

## Data Availability

The data are not publicly available because they contain identifiable patient photographs but are available from the corresponding author upon reasonable request.
